# Presenting quantitative information about decision outcomes: a risk communication primer for patient decision aid developers

**DOI:** 10.1186/1472-6947-13-S2-S7

**Published:** 2013-11-29

**Authors:** Lyndal J Trevena, Brian J Zikmund-Fisher, Adrian Edwards, Wolfgang Gaissmaier, Mirta Galesic, Paul KJ Han, John King, Margaret L Lawson, Suzanne K Linder, Isaac Lipkus, Elissa Ozanne, Ellen Peters, Danielle Timmermans, Steven Woloshin

**Affiliations:** 1 Primary Health Care, School of Public Health, Room 321b, Edward Ford Building (A27), University of Sydney, NSW 2006, Australia; 2 Department of Health Behavior & Health Education, School of Public Health, Department of Internal Medicine, School of Medicine, and Center for Bioethics and Social Sciences in Medicine, University of Michigan, 1415 Washington Heights, Ann Arbor, MI 48109, USA; 3 Cochrane Institute of Primary Care and Public Health, School of Medicine, Cardiff University, Neuadd Meirionnydd, Heath Park, Cardiff CF14 4YS, UK; 4 Harding Center for Risk Literacy, Max Planck Institute for Human Development, Lentzeallee 94, 14195 Berlin, Germany; 5 Center for Adaptive Behavior and Cognition, Max Planck Institute for Human Development, Lentzeallee 94, 14195 Berlin, Germany; 6 Center for Outcomes Research and Evaluation, Maine Medical Center Research Institute, 509 Forest Avenue, Portland, ME 04101, USA; 7 Department of Family Medicine, University of Vermont College of Medicine, 235 Rowell, 106 Carrigan Drive, University of Vermont, Burlington, Vermont 05405, USA; 8 Department of Pediatrics, Children’s Hospital of Eastern Ontario, University of Ottawa, 401 Smyth Road, Ottawa, Ontario, K1H 8L1, Canada; 9 Department of General Internal Medicine, The University of Texas MD Anderson Cancer Center, 1515 Holcombe Blvd, Houston, TX 77030, USA; 10 Duke University School of Nursing, 307 Trent Drive, Durham, NC 27710, USA; 11 Department of Surgery and Institute for Health Policy Studies, University of California, San Francisco, 3333 California St. Suite 265, San Francisco, CA 94143-0936, USA; 12 Department of Psychology, Ohio State University, 235 Psychology Building, 1835 Neil Avenue, Columbus, OH 43210, USA; 13 Department of Public and Occupational Health, EMGO Institute for Health and Care Research, VU University Medical Center, Van der Boechorststraat 7, 1081 BT Amsterdam, The Netherlands; 14 Departments of Medicine and of Community & Family Medicine and The Dartmouth Institute for Health Policy & Clinical Practice at the Geisel School of Medicine at Dartmouth and the VA Outcomes Group, VA Medical Center, 215 North Main Street, White River Junction, VT 05009-0001, USA

## Abstract

**Background:**

Making evidence-based decisions often requires comparison of two or more options. Research-based evidence may exist which quantifies how likely the outcomes are for each option. Understanding these numeric estimates improves patients’ risk perception and leads to better informed decision making. This paper summarises current “best practices” in communication of evidence-based numeric outcomes for developers of patient decision aids (PtDAs) and other health communication tools.

**Method:**

An expert consensus group of fourteen researchers from North America, Europe, and Australasia identified eleven main issues in risk communication. Two experts for each issue wrote a “state of the art” summary of best evidence, drawing on the PtDA, health, psychological, and broader scientific literature. In addition, commonly used terms were defined and a set of guiding principles and key messages derived from the results.

**Results:**

The eleven key components of risk communication were: 1) Presenting the chance an event will occur; 2) Presenting changes in numeric outcomes; 3) Outcome estimates for test and screening decisions; 4) Numeric estimates in context and with evaluative labels; 5) Conveying uncertainty; 6) Visual formats; 7) Tailoring estimates; 8) Formats for understanding outcomes over time; 9) Narrative methods for conveying the chance of an event; 10) Important skills for understanding numerical estimates; and 11) Interactive web-based formats. Guiding principles from the evidence summaries advise that risk communication formats should reflect the task required of the user, should always define a relevant reference class (i.e., denominator) over time, should aim to use a consistent format throughout documents, should avoid “1 in x” formats and variable denominators, consider the magnitude of numbers used and the possibility of format bias, and should take into account the numeracy and graph literacy of the audience.

**Conclusion:**

A substantial and rapidly expanding evidence base exists for risk communication. Developers of tools to facilitate evidence-based decision making should apply these principles to improve the quality of risk communication in practice.

## Background

Health decisions often require patients and clinicians to compare and choose among two or more options. The chosen path may bring with it a number of benefits and harms for the individual patient. Informing these choices with the best available evidence from scientific research is desirable and, where available, outcomes should be provided that have been quantified through research [1,2]. For both written and verbal information, patients have a more accurate understanding of risk if probabilistic information is presented as numbers rather than words, even though some may prefer receiving words [3].

Patient decision aids (PtDAs) can be an effective strategy for integrating research evidence with patient values and other factors to facilitate greater patient involvement, improve decision quality, and increase knowledge about decision options [4]. Sixteen out of the 86 trials in the Cochrane Collaboration review of PtDAs for treatment and screening decisions measured the effects of including numeric estimates on patients’ understanding [4]. Presenting numeric estimates within a PtDA significantly improved the accuracy of risk comprehension (RR 1.7, 95% CI 1.5 to 2.1), compared with not receiving numeric estimates, with larger effect size when estimates were presented as numbers, versus describing them in words.

### A theoretical rationale for evaluating patient decision aids on this quality standard

The argument outlined above serves, in effect, as a broad justification for considering *“presenting quantitative information about decision outcomes in PtDAs”* as an important quality standard when evaluating a PtDA. However, although the inclusion of numeric risk estimates in PtDAs appears to be an effective strategy for promoting informed decision making, many important questions about risk communication remain. Exactly how should numeric risk estimates be represented in order to maximize patient understanding? What are the essential elements of effective risk communication, and does empirical evidence support particular methods or “best practices” for representing and communicating numeric risk estimates using PtDAs?

### Purpose

While we were updating the International Patient Decision Aid Standards (IPDAS) Collaboration’s original Background Document, our working group developed a wide-ranging review of current knowledge in the field of risk communication, including relevant evidence from the broader science and social science literature. The purpose of this paper is to summarize our insights into the “state of the science” about the presentation of quantitative information about decision outcomes, and, in doing so, to focus on communication issues that are likely not only to have practical application in the development of PtDAs but also to inform the further development of the quality standards.

## Empirical evidence

### Method

Since the literature on risk communication is so vast, this evidence summary was developed by expert consensus. Our focus was to provide clear guidance for PtDA developers regarding design issues for which substantial research evidence exists and to identify those design problems remaining to be resolved. Thus, since the purpose of this review was to provide guidance about risk communication generally, not to answer a focussed question, a systematic review was not feasible or appropriate.

The expert international working group was formed by inviting key authors in the field. The fourteen individuals who agreed to participate were drawn from North America, Europe and Australasia. Several had participated in writing the first version of this material in the IPDAS Collaboration’s 2005 Background Document.. As a first step, members of the group identified major issues in communicating quantitative information for PtDA development. This was achieved through an iterative and interactive online discussion process and drew heavily upon the combined expertise of the authors.

Each member of the working group assisted in drafting at least two of the issue-focused sections and worked closely with at least one other author in this task. Each team drew upon their collective expertise to define current best practices for communicating probabilities in PtDAs and to provide illustrative research findings in support of their recommendations.

The entire working group then provided input and peer review to the full draft document, resolving disagreements through additional debate and discussion to reach consensus. The group also took time to clarify a number of definitions to address some of the confusion arising from terminology used in the various risk communication domains. These definitions are listed in Table 1.

**Table 1:** Terms and definitions in risk communication

Term	Definition
**Simple frequency format**	Expresses the event rate as an integer with an appropriate denominator (e.g. x in 100)

**Simple percentage format**	Expresses the event rate as a percentage (e.g. x%)

**Natural frequency format**	The term ‘natural frequencies’ was proposed for estimating the probability arising from a joint occurrence of events (e.g. the probability of having breast cancer given an abnormal mammography result). Natural frequencies preserve the base rate of the outcome (e.g. breast cancer) and report the ‘actual’ or ‘natural’ number of people having a particular outcome (e.g. having a positive test result). An example would be “Out of every 10,000 people, 30 have colorectal cancer. Of these, 15 will have a positive haemoccult test. Out of the remaining 9970 people without colorectal cancer, 300 will still test positive. How many of those who test positive actually have colorectal cancer? Answer: 15 out of 315”

**Conditional probabilities**	An alternative representation of this information is the conditional probability format. For example: “The probability of having colorectal cancer is .003%. Of people who have the cancer, 50% get a positive test result. Of people who do not have cancer, 3% will nevertheless test positive. What is the probability that a person who tests positive has colorectal cancer? Answer: 4.8%”.

**Bayesian reasoning**	Infers the post-probability of outcome from the prior probability and a likelihood function.

**Tailored health communication**	Refers to providing information to a person based on characteristics that are unique to that person. It is assumed that tailored messages are perceived as more relevant to an individual and are therefore better processed and understood. Tailoring information using an individual’s specific risk factors might likewise increase people’s involvement with the information and lead to a better understanding.

**Aleatory uncertainty**	It is concerned with the randomness or indeterminacy of future events.

**Epistemic uncertainty**	On the other hand, this is the lack of knowledge needed to predict future outcomes, also known as “ambiguity” and is concerned with the lack of reliability, credibility, or adequacy of risk information. A primary example is imprecision in risk estimates which are typically expressed by confidence intervals.

**Pictographs (sometimes called icon arrays)**	They are visual graphic display formats which aim to represent the size of both the numerator and denominator in the one diagram. In other words, they show the part-whole relationship. Examples include systematic ovals, 100 face or human figure diagrams and displays where event icons are scattered rather than grouped.

**Numeracy**	It is the ability to understand and apply mathematical concepts.

**Patient narratives**	Stories, also called testimonials, about individuals’ experiences or health outcomes, usually told from a first-person perspective.

### Results

We identified eleven major risk communication issues involved in the presentation of probabilities and related risk information: 1) Presenting the chance an event will occur; 2) Presenting changes in numeric outcomes; 3) Outcome estimates for test and screening decisions; 4) Numeric estimates in context and with evaluative labels; 5) Conveying uncertainty; 6) Visual formats; 7) Tailoring estimates; 8) Formats for understanding outcomes over time; 9) Narrative methods for conveying the chance of an event; 10) Important skills for understanding numerical estimates; and 11) Interactive web-based formats.

#### Presenting the chance an event will occur

**Figure 1:**
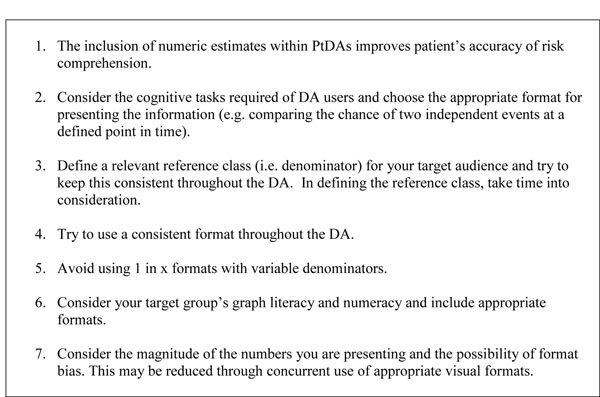
Guiding principles for including numeric estimates in decision aids

In Figure 1, we outline some guiding principles for including numeric estimates in decision aids. Below, we discuss these principles in detail.

Suitable formats for presenting numeric chances depend on the nature of the task [5]. When the task is to present the chance of a single event, simple frequency formats that include a number and time interval (such as “Every year 10 in 100 people with pre-diabetes develop diabetes”), or simple percentage formats (such as “Every year 10% of those with pre-diabetes will develop diabetes”) are more transparent than formats such as “The chance of developing diabetes is 10%”. The last statement is problematic because it does not specify the “denominator” (i.e., the reference class, as in, for example, “10% of all pre-diabetics in one year”). Without a clear description of to whom this estimate refers, people might impose their own erroneous interpretations such as “I only have a 10% chance of developing diabetes in my lifetime” [6]. Similarly, when patients who take fluoxetine for mild depression hear from their doctor that there is a “30-50% chance of developing a sexual problem such as impotence or loss of sexual interest,” some may think this means they will have problems in 30% of their own sexual encounters. The “denominator” or reference class used by the doctor is “patients on fluoxetine”, but the denominator used by the patient is “their own sexual encounters” [7].

There is also some evidence that risks presented in simple frequencies are perceived as higher than when they are presented in their equivalent percentage value, especially in patients with lower numeracy [8] and (possibly) when smaller percentages are presented [9]. Given this potential format bias, one should be careful when comparing results of studies that have used different formats (percentages or simple frequencies). Formats should aim to be consistent throughout a PtDA (see below). Providing simple frequency AND percentage appears to add no advantage [9], and there is strong evidence that “1 in x” formats with variable denominators are more difficult to understand and elevate risk perceptions[10,11]. They should be avoided for all tasks.

*1.* In summary, it is most important when presenting the chance of a single event to clearly define the denominator or reference class over time. Percent *or* simple frequency formats can be used for presenting the chance of a single event. However, in deciding which one to use, consider what other information needs to be presented and what the purpose of the PtDA is, overall, so there is format consistency throughout. Visual formats may also help to reduce bias (see *Formats for understanding outcomes over time*, below).

When the task is to compare the chance of occurrence of two or more independent events (e.g., the chance of symptom relief with drug A compared with placebo), formats that express the chance of an event using one number, such as percentages, work better than simple frequencies involving more than one number, such as 1 in 100 [9]. If using simple frequencies such as 1 in 100, one should use the *same* denominator (e.g., 1 in 100 versus. 2 in 100) as these are easier to compare than frequencies using different denominators (e.g., 1 in 100 versus. 1 in 50) [8,10]. Consistent denominators should always be used. When choosing the size of the denominator, smaller numbers (e.g., 100) are easier to understand and remember than larger numbers (e.g., 10,000) [12]. There has been discussion about whether people find percentages less than one (e.g., 0.1%) more difficult to understand than the equivalent simple frequency (e.g., 1 in 1000) [9,10]. However, this problem may reflect difficulty manipulating decimal points (e.g., asking someone to represent 1 in 1000 as a percentage) rather than a comprehension problem [9].

In summary, percentages (e.g., x %) may have an advantage over a simple frequency format (e.g., x in 100) for comparing the chance of occurrence of two or more independent events. As mentioned before, it remains important to clearly define the denominator or ‘reference class’ and to aim for a consistent format throughout the PtDA taking into account the information and tasks required.

However, other formats are more suitable for tasks that involve presenting changes in numeric outcomes (see *Presenting changes in numeric outcomes*, below) and conveying the frequency of joint occurrences of two or more *dependent* events, such as the conditional probability that a person with a positive test result has the disease (e.g., see *Outcome estimates for test and screening decisions*, below).

#### Presenting changes in numeric outcomes

Most efforts to communicate changes due to interventions (i.e., treatment effects) or across time (e.g., improvements of health) use either side-by-side total risk presentations or difference presentations. Difference presentations depict the change in risk and can influence risk perceptions through framing effects. Research has shown that relative risk presentations (e.g., “30% lower risk”) tend to magnify risk perceptions and decrease understanding, compared to absolute risk presentations (e.g., “the risk is lower by 5 percentage points”)[13,14]. Number needed to treat (NNT) is sometimes used, but several studies suggest that this format is poorly understood by patients and may increase the perceived effect of treatment [15].

A variant is to present incremental risk (absolute risk increase) after a “baseline” total risk level has been shown. This approach emphasizes the size of the change relative to the size of the total risk, and was shown to lower risk perceptions [16]. Such language (e.g., “5 more women get…”) was incorporated into the Schwartz et al. drug facts box [17], and used in decision tools such as Adjuvant! A PtDA trial suggested that incremental risk language works best when accompanied by visual displays [18]. In particular, when the baseline risks are small, relative risk reductions are perceived to be larger than absolute risk reductions [13,19,20].

The framing of outcomes in terms of losses or gains has been shown to affect people’s choices [21]. Framing outcomes in terms of potential gains (e.g., the chances of survival) often generates risk-averse choices, whereas framing outcomes in terms of potential losses (e.g., the chances of death) often generates risk-seeking choices. In clinical situations, the effects of the framing of outcomes as losses or gains tend to vary across situations [22]; the variable effect of different frames of risks or rates is due to emphasizing different aspects of the information.

In summary, when presenting changes in rates, absolute risks should be given either in percentages or simple frequencies, and if possible along with the absolute risk increase (or decrease). If frequencies are used, the denominators should be equal.

#### Outcome estimates for test and screening decisions

A number of studies and a recent Cochrane Collaboration review [13,23] have shown that natural frequencies are better than conditional probabilities where events are connected. It is unclear whether people use Bayesian reasoning when making screening decisions, but natural frequency formats are still proposed as the best way to help people understand these kinds of estimates [24,25]. So, if a PtDA requires people to calculate the probabilities associated with jointly occurring events, then a natural frequency format would be preferable to conditional probabilities.

However, screening can also be viewed as an “intervention” that has an effect (e.g., reducing death from colorectal cancer). Rates of death from a particular cancer with and without screening are actually the chances of independent events. As noted earlier, there may be some advantage to presenting such information in a percentage format, but cancer incidence and mortality rates are usually low in the general population and possible format biases due to small numbers need to be considered (see *Presenting the chance an event will occur*, above). Similarly, the chance of having a disease if your test result is positive can be thought of as the “post-test probability”, and some would suggest this could be calculated on behalf of the patient and presented in a percentage (1%) or simple frequency format (e.g., 1 in 100). Thus, we recommend that PtDA developers consider both the nature of the task required and the other information that needs to be conveyed in the same document. It is important to clarify what the reference class is (e.g., women aged 50 who are having biennial mammography over 10 years) and to keep the denominator constant. Once again, “1 in x” formats should be avoided as they consistently perform worse.

The current IPDAS criteria recommend that screening PtDAs include estimates of: 1) disease with and without screening; 2) false positives; and 3) false negatives. The updated Cochrane Collaboration review of PtDAs includes 34 trials about screening and test decisions [26]. Five of these trials measured the accuracy of risk perception [27-31]. Four of these reported significantly improved risk perception [27-30] regardless of whether accurate risk comprehension was measured as numbers [27,28] or as gist-based risk comprehension in words [29,30]. Four of the trials also included quantitative estimates in accordance with the IPDAS criteria recommendations. Three of the PtDAs were available and all used different formats for numerical outcomes. None provided a head-to-head comparison of formats. Given the lack of head-to-head format comparison in these trials, we recommend applying the principles outlined in this chapter which are based (where possible) on comparative research.

Our review confirms that, in screening PtDAs, the application of IPDAS criteria about the presentation of quantitative estimates of screening outcomes improves the accuracy of risk perceptions.

#### Numeric estimates in context and with evaluative labels

To help users get perspective on the risk of disease, PtDA developers should consider including contextual information when feasible. Context is particularly important for PtDAs about disease prevention or cancer screening, in which the benefit is a reduction in disease specific mortality. One way to provide context is to provide the chance of death over the next 10 years from the disease under consideration (where possible according to age, smoking status, and other reliable risk factor information), as well as the chance of dying from other major causes and from all causes combined [32].

Directly interpreting the meaning of numeric information (e.g., telling patients how good or bad a 9% risk is) can also have a substantial influence on how patients use that information. This is because patients often do not understand the meaning of unfamiliar numbers without additional help, and, without meaning, information tends not to be used in subsequent decision making. In one series of studies, providing evaluative labels for numeric quality-of-care information (e.g., telling decision makers that the numbers represented “poor” or “excellent” quality of care) resulted in greater use of this information in judgments and less reliance on an irrelevant emotional state among the less numerate [33]. In another study, evaluative labels for test results (that a patient’s test was “positive” or “abnormal”) induced larger changes to risk perceptions and behavioural intentions than did numeric results alone [34]. The appropriateness of these changes, however, can be unclear in health contexts, and evaluative labels should be applied carefully.

#### Conveying uncertainty

Numeric risk estimates ultimately represent evidence-based, mathematical expressions of uncertainty about the future. The uncertainty expressed by risk estimates can be divided into two principal types: 1) “aleatory” or first-order uncertainty that reflects the randomness or indeterminacy of future events, and 2) “epistemic” or second-order uncertainty, otherwise known as “ambiguity,” that reflects limitations in the reliability, credibility, or adequacy of risk information. An understanding of each of these uncertainties is arguably essential for informed decision making. However, the optimal methods and outcomes of communicating these uncertainties to patients are only beginning to be understood [35].

The communication of aleatory uncertainty has been examined in a small number of studies for both textual and novel visual methods of representing randomness in decision support interventions (e.g., icon arrays displaying affected individuals in a scattered rather than clustered manner) [36-40]. Available evidence suggests that these methods have no significant effect on risk perceptions, although evidence is lacking regarding their effects on patients’ understanding of uncertainty. In one study, however, the communication of randomness was associated with greater subjective uncertainty about estimated risk [41]. The communication of epistemic uncertainty has been examined in a small number of studies using confidence intervals to communicate ambiguity in probability estimates. These studies have shown that communicating ambiguity has little effect on risk perceptions, although it increases patient worry [41,42], and these effects appear to be moderated by representational method (visual vs. textual) and individual differences (e.g., dispositional optimism) [39,41]. Evidence is limited and mixed regarding the extent to which confidence intervals are understood by patients [43,44] and how they influence perceptions of the credibility of probability estimates [38,45]. Furthermore, the effects of communicating both epistemic and aleatory uncertainty on real medical decisions have not yet been evaluated.

The communication of ambiguity has been evaluated more fully outside health care. Numerous studies in behavioural decision research have shown that ambiguity leads to avoidance of decision making and pessimistic risk perceptions and affective responses (worry, distress) related to choice outcomes—a phenomenon known as “ambiguity aversion” [46-49]. However, most studies have examined hypothetical rather than real decisions.

In summary, evidence on the optimal methods and outcomes of conveying uncertainty is limited but growing. Novel representational methods have been developed to communicate both randomness (aleatory uncertainty) and ambiguity (epistemic uncertainty); these methods may be useful to incorporate in PtDAs along with estimates of risk magnitude. However, the communication of uncertainty can be psychologically aversive. More research is needed to determine both the optimal representational methods and effects of communicating uncertainty on patient perceptions, understanding, and decision making.

#### Visual formats

Presenting event rates with visual aids such as pictographs (also called icon arrays), bar charts, or flow diagrams may aid accurate understanding of probabilities. Visual displays can help reduce several biases, such as denominator neglect [50], framing effects [51,52], and the undue influence of anecdotes [53]. They also can aid the comprehension of more complicated concepts such as incremental risk [18]. Graphs that clarify sub-set relationships (e.g., Venn diagrams, Euler circles) can lead to better judgements, for instance in Bayesian reasoning tasks [54,55]. Others believe graphs help, but for different reasons [56]. However, there has been some evidence that graphs can affect peoples’ tendencies to overestimate low probabilities and underestimate high probabilities – the magnifier effect [57]. Others have shown the opposite effect (i.e., less overestimation) on low probabilities and no effect on high [58].

Although the use of visual displays is often recommended as an aid to interpretation for numerical data [59,60], one important caveat is that people vary in their ability to extract data and meaning from visual displays. Galesic & Garcia-Retamero developed a graph literacy scale that predicts who actually profits from visual displays [61,62]. For example, visual displays are helpful for understanding statistical information about health for people with low numeracy [63,64] yet people who lack graph literacy may be better off with just numbers [65].

Graphs have sometimes been shown to be better able to convey the essential aspects of the information (i.e., “gross-level information”) [66], bottom line meaning, or gist [67], whereas numerical representations may better convey more precise aspects of the information (i.e., “detailed-level information” or verbatim) [66,68]. Thus, a potential weakness of visual displays is that people may focus more on the pattern of data rather than the precise values, unless that is the main objective. Furthermore, some graphs are better suited for certain tasks (e.g., line graphs for trends over time, bar graphs for comparison across groups) [69,70].

Graph type and formatting have an effect on comprehension and behaviour. For instance, one study showed that the formats that are perceived most accurately and easily by patients are vertical bars, horizontal bars, and pictographs. Preferred graphs do not necessarily lead to better performance than non-preferred graphs. Furthermore, pie charts and pictographs with randomly-distributed ovals lead to slower and less accurate estimates [66]. Another study showed that different visual formats supported gist versus verbatim knowledge [68]. Enhancing accuracy in estimates can be aided by displaying only the most crucial elements [71,72], as well as by using icon arrays (blocks or stick figures) that are arranged as groups in a block rather than being scattered randomly -- the latter of which is useful to convey the concept that events (e.g., who is afflicted by disease) occur at random [73].

Finally, it has been shown that visual aids are most effective for comprehension when the entire population at risk is shown rather than only depicting sick people, for instance [62]. In addition, for conveying small probability events (e.g., less than 1%), graphical displays (e.g., bar charts) that show only the number of people affected (i.e., foreground information) leads to greater risk aversion (e.g., greater willingness to pay for an improved product) than graphic displays that show part-whole relationship by including the total population or those not affected) (i.e., background) [74,75]. It is still unclear whether icon-arrays (which show the part-whole relationship by including the full denominator) result in more accurate risk perception than icon-based displays (which do not have the full denominator).

A recent study in adults with lower education and literacy [76] found that numerator size was an important factor when presenting the changes in numeric outcomes for events out of 1000. Where the outcome is <100/1000, pictographs were better understood and processed more quickly than bar charts, particularly if the difference between event rates was small. These 1000 denominator pictographs stacked the icons along the long axis beside a referent scale to support easier reading. However, for more common outcomes ( >100/1000), bar charts were better, possibly because the icon arrangement was more complicated. In addition, the role of shading in processing the part-to-whole relationship of icon arrays is still not well understood.

In summary, visual displays can be a powerful tool to convey health-related statistical information, especially for people with higher graphical literacy and among those who have problems with understanding and applying numbers. However, some caution is warranted as visual displays may not be intuitively understood by everyone. They can be used to represent statistical information transparently, but they can also be misused and misrepresent statistical information [77]. Overall, all visual aids should be pilot tested for understanding (not simply preferences), and developers should take care to avoid using misleading images (such as graphs with misleading scales) or using different scales within the same PtDA. Finally, the field still needs a more systematic theoretical understanding of why, when, and for whom visual displays are effective [69]. Such theories could help to translate the growing research in graph cognition and design [78,79] into practical advice for risk communicators.

#### Tailoring estimates

To date, the effects of tailoring health risk information on improving health decision making appear mixed. Limitations in research quality and heterogeneity in outcome measures make drawing firm conclusions about effective strategies difficult. A meta-analytic review showed that tailored print messages about health have been effective in stimulating health behaviour change, but the effect size is small and depends on the variable that is used for tailoring [80]. The effect of tailoring was modified by the type, visual layout and length of the printed material, type of behaviour (more effective for preventive behaviours) and by demographic factors [80,81]. Tailored print messages have been shown to increase uptake of mammography screening [81] and pap testing [80,81]. A review by Albada et al. showed that information tailored to an individual’s risk factors increased risk perception and resulted in better knowledge compared to generic information [82].

Results also are mixed with respect to the effect of tailored health messages on behaviour—for example, on cancer screening. Tailoring by behavioural constructs seems to be effective, while there was limited evidence of the effectiveness of information tailored by risk factors only, in particular for cancer screening. Bodurtha et al. also found that a ‘brief (tailored) intervention’ regarding mammography adherence did not change behaviour [83]. No significant differences existed in mammography intentions, actual uptake, clinical breast examination, or self-examination between intervention and control study arms. However, among those who were most worried, mammography rates in the intervention group were higher. Thus, individual characteristics, such as worry about breast cancer and educational status, may modify the effects of tailored health messages.

Because most studies on tailoring health risk information were done for cancer screening, not much is known about the effect for other decisions. More insight is needed into *why* personalized risk messages might be better understood and whether they are relevant for other kinds of health decisions.

#### Formats for understanding outcomes over time

Choices of how to display long term outcomes to improve understanding of risk are challenged by the difficulty in obtaining accurate relevant long-term outcome estimates of benefit and risk [84]. Randomized controlled trials and systematic reviews usually represent a few years of follow up at most. Yet to make an informed decision, patients and physicians are often interested in longer term outcomes. Observational studies can provide longer-term data but are prone to selection bias and confounding. An additional bias is the tendency for trials to aggregate short and long term mortality which leads to inaccurate estimates if hazard ratios are not constant over time [85]. These methodological problems are beginning to be addressed by newer risk modeling approaches [86-89].

When data are available, formats used to improve patient understanding of outcomes over time include: (a) the chance of a specific outcome at a single point in the future; (b) chance of an outcome at multiple points in the future; (c) mortality or survival graphs showing risks over time; (d) cumulative future or lifetime chance of an outcome; and (e) rate of occurrence of an outcome that is likely constant over time.

Showing the chance of a specific outcome at a single point in the future has the advantage of simplicity of presentation and calculation from available randomized trials or cohort studies. Examples of this approach are the 10 year risk of cardiovascular disease used in estimates of risk and benefit of cholesterol medications [90] and the risk in 3-5 years of precancerous changes on pap smear or genital warts related to HPV vaccine [91]. This method has also been used with multiple points in the future. Examples include presenting the risk of having to have repeat bypass surgery at 5 years and 10 years after the initial procedure [92], and expected deaths after lung transplantation for cystic fibroses shown at 1 month, 1 year, 3 years, 5 years, and 10 years [93].

Survival and mortality graphs are commonly used in presenting research studies and have been used to relay information to patients. However, patients’ interpretation of these graphs may be susceptible to various biases. When web-users were shown survival graphs for a hypothetical disease and treatment, they based their perceptions of treatment effectiveness on visual differences in these graphs [94]. When a longer duration of data was shown, people perceived larger differences in risk even when the magnitude of risk reduction was identical. Mortality graphs may be more temporally consistent [95], but less well understood by patients [96]. Given these findings and current limitations in evidence, a balanced approach using both survival and mortality graphs may be prudent until more information is available [97]. A study presenting treatment options for esophageal cancer showed most patients understood graphical representations of even complex multidimensional patient-reported outcomes [98].

Another common format for representing outcomes over time are estimates of the cumulative chance of an event during a whole lifetime, although these can be difficult for people to understand [99]. Nevertheless, this method is commonly used—e.g., in describing cancer risk in patients with BRCA gene mutations [100]. People are also often shown the cumulative chance of an event over a certain period of time into the future (e.g., 10 years); for example osteoporosis treatment [101] and hormone replacement therapy in menopause [102]. Cumulative risk over time is also used in PtDAs without an explicit endpoint when describing probabilities of outcomes after a specific event or intervention. Examples include comparisons of outcomes of Achilles tendon rupture with and without surgery [103], and of cardiac resynchronization therapy in heart failure [104]. Rates are also used in conditions likely to have a relatively constant risk over time. An example is birth control and the annual risk of pregnancy with a specific method [105].

Although PtDAs providing quantitative risk information have been shown to increase accuracy of risk perceptions [26] and to promote knowledge and agreement between values and choices [26], there are no trials examining different formats for representing the risk of outcomes over time.

#### Narrative methods for conveying the chance of an event

Individual patient narratives (i.e., stories of what one person experienced or did, which are sometimes referred to as “patient testimonials”) are sometimes provided in PtDAs as a complement to statistical information about risks. These stories can provide rich detail about what it is like to experience a health condition or a particular outcome of treatment. However, the provision of narratives has been shown to influence perceived vaccination risk and intentions [106]. In one study, narratives decreased the perceived chance of adverse events but increased the perceived severity of adverse events. Narratives also influenced vaccination intention even after controlling for the perception of vaccine riskiness. In the same study, the nature of the presented information (emotionality, richness) was also varied to assess the impact on risk perception and showed that the highly emotional narratives had a greater impact on perceived risk although the richness of the narratives did not.

Other studies have shown that patient testimonials influence treatment choices. In one study, participants receiving a disproportionate number of negative testimonials for surgery were less likely to choose surgery compared to participants receiving equally positive and negative examples for surgery [107]. In Ubel’s second study, participants receiving no testimonials were most likely to choose bypass surgery (58%), compared to those receiving a proportionate number of testimonials (37%) and those receiving a disproportionate number of testimonials (34%). In this case, the testimonials significantly reduced the choice of the most effective but invasive and risky intervention.

Another study tested whether the use of a quiz or pictograph lessened an individual’s reliance on anecdotal evidence for angina treatment (bypass surgery or balloon angioplasty) [53]. They found that, when statistical information was reinforced with pictographs and quizzes, anecdotes had no significant effect on treatment decisions. The same authors also found pictographs were the active ingredient that lessened the effect of anecdotes. This finding would argue for avoiding narratives without statistical information.

In summary, using narratives to present benefit and risk information may increase perceptions of risk severity, decrease the ability to accurately recall risk probabilities, and influence treatment choice. The relative number of narratives used can also influence decision making. However, narratives vary in their purpose, content, and emotional balance[108], and some types of narratives may be more or less likely to bias risk perceptions than others. For example, in principle, narratives that report outcomes should influence likelihood perceptions more than those which describe experiences or decision processes [108]. Because of the potential that narratives might have unintended effects on risk communications, we suggest (a) that narratives should be used with caution until research better clarifies their effects, both positive and negative, and (b) that developers be more cautious about using narratives when attempting to present unbiased information for informed decision making than when attempting to be persuasive and promote behaviour change. If narratives are used to present benefit and risk information, they should be accompanied by statistical information in pictograph form. Graphical representations of risk may reduce the effect of narratives. However, it seems likely that information included in narratives is likely to influence the ways individuals either search for and/or process information, which may make them either useful or counterproductive when included in interventions designed to facilitate good decision making.

#### Important skills for understanding numerical estimates

Numeracy is the ability to understand and apply mathematical concepts. It can have considerable effect on the use and interpretation of numerical estimates. Higher numeracy can facilitate computations, the interpretation of numbers, information seeking, depth of processing, memory for numerical information, and trust in numerical formats, leading to improved risk comparisons, risk estimates, and value elicitations [33,109-113]. Lower numeracy is associated with overestimation of risk probabilities [114,115], higher susceptibility to factors other than numerical data (e.g., framing, mood states, labels used to interpret quantitative results and feedback from others) [33,116], insensitivity to variations in risk magnitude (especially when multiple numbers are shown at once) [117], and greater denominator neglect [64,118]. In particular, people with lower numeracy are less likely to derive affective meaning from numbers and are more influenced by affective considerations from non-numerical aspects of the task [119].

Levels of numeracy in the general population are relatively low. For example, a national survey showed that one-quarter of the general US population cannot say whether 1 in 10, 1 in 100, or 1 in 1000 represents the largest risk of getting a disease; 30% cannot transform 20 in 100 to a percentage; 40% cannot say what is 1% out of 1000; and about 75% cannot transform 1 in 1000 to a percentage [120]. Greater numeracy is associated with higher education, younger age, and male gender [109,110], although none of these characteristics guarantee high numeracy. For example, even highly educated people can be less numerate [121] and research has demonstrated that numeracy can be more predictive of comprehension and decisions than education and other demographic variables [122-125].

Numeracy is relatively distinct from other aspects of health literacy, general intelligence, and working memory [122,126,127] and can be acquired through instruction and deliberative practice [128]. Educational systems that focus on mathematics and science education from an early age may contribute to higher numeracy levels in the general population [120,129]. Short interventions aimed at improving patients’ numeracy and consequently their risk understanding might be useful, but, at present, research regarding the feasibility or efficacy of such interventions is lacking.

Higher numeracy, however, does not preclude the need for well-designed PtDAs. Patients need at least basic numeracy but the communicators need to be aware of and use an evidence-based approach to present information in ways that are simple and clear. Even the highly numerate can misunderstand inconsistent or difficult information formats such as those involving unequal denominators, unspecified reference classes, or conditional probabilities. Furthermore, situational factors that are often present in medical decision making, such as time pressure and high levels of stress, can make the processing of risk related information difficult even for highly numerate people [109]. In addition, as individuals age, their numeracy levels tend to decline [110,130]; this is important because older adults tend to make a disproportionate number of medical decisions relative to healthier younger adult populations. Finally, more numerate individuals may be *more* susceptible to some biases than the less numerate (e.g., evaluating a possible gain as more attractive when put into a context of a possible small loss than when presented alone [116,131]. As a result, numeracy will usually help but sometimes may hinder patients’ abilities to use the most important dimensions in a decision. Unintended consequences of higher numeracy have not been considered yet in health contexts, but, ultimately, may be necessary to understand.

When designing a PtDA, understanding the abilities of prospective users can help in designing presentation formats that will maximize comprehension and use of important information in PtDAs. For example, among those with lower numeracy *and* sufficient graph literacy, visual displays can improve understanding [62,65,132,133]. A number of measures of patients’ abilities relevant for understanding of risks have been developed in recent years (e.g., objective numeracy [121,134,126,136]; subjective numeracy [137]; graph literacy [61]; other aspects of the health literacy [138,139]. Unfortunately, with few exceptions [71,111], studies of how numeracy and graph literacy influence use and interpretation of numerical data in PtDAs are lacking, and hence sorely needed. In the absence of such data, it is difficult to assess whether tailoring PtDAs to patient’s abilities would result in any improved outcomes. Given that assessing patients’ numeracy and graph literacy in busy clinic settings may be difficult, it may be preferable to design PtDAs that benefit patients across the whole continuum of abilities, and to teach medical or other personnel to help patients make sense of the information.

#### Interactive web-based formats

The increasing prevalence of computers, tablets, and mobile devices creates new opportunities for interactive, web-based formats for communicating probability information. The literature in this area is sparse, and we are aware of no published studies that have examined use of such tools in actual PtDAs. Several experimental studies suggest, however, that web-based formats offer both opportunities and pitfalls. For example, in one study, participants presented with a treatment scenario were better calibrated in their perceptions of medication side effects when they created a bar graph of the risk instead of just viewing one [140]. Another study found that a web-based, game-like, interactive risk graphic in which participants clicked in a matrix until they uncovered a risk event had the effect of reducing disparities in risk perceptions between more and less numerate participants [141]. Such exercises could be seen as methods to increase patients’ active processing of risk information, which may lead to improved risk understanding. Indeed, the game-like interactive task elicited stronger emotional responses [40].

However, there are also considerable grounds for concern about interactive risk graphics. In 2002, Tversky, Morrison, and Betrancourt reviewed the literature on animated graphics of all types and noted that “the research on the efficacy of animated over static graphics is not encouraging” [142]. More recently, research participants who used an interactive pictograph applet to visually graph provided risk numbers had significantly worse knowledge and made poorer decisions than participants who viewed static graphs [143].

Even without interactivity, animated graphics can use motion cues to reinforce gist messages. However, the evidence here is also mixed. One study found a dynamic scattered icon display increased recipients’ subjective uncertainty about a risk [39]. Another study tested various types of animation in both grouped and scattered icon displays and found that they failed to improve participants’ ability to identify a dominant treatment option and sometimes significantly impeded performance [144].

In short, interactive web-based risk communication formats allow educators to use additional cues in risk communications. However, evidence is lacking to determine whether the techniques allowed by new technologies provide a net positive experience. Preliminary evidence suggests that, unless the motion cues reinforce the most critical gist message (e.g., the accumulation of risk over time), there remains significant risk that interactive or animated formats may degrade knowledge versus evidence-based static formats.

## Discussion

The science around risk communication is expanding rapidly, and there is good evidence that patients have a better understanding of risk if outcomes are presented as numbers. Yet there is an emerging awareness that how risk information is provided can improve people’s understanding or bias their risk perceptions. We highlight in Table S1 (Additional file 1) our key messages regarding each of the eleven major risk communication issues involved in the presentation of probabilities and related risk information.

For example, it is increasingly clear that different formats work better for different tasks, and PtDA developers should consider what cognitive tasks are required for each decision. Our review also makes it clear that it is important to include contextual information and to consider the numeracy and graph literacy skills of the PtDA users. Some format biases can be reduced by concurrent use of visual formats such as graphs and there are some formats which should be avoided as they consistently perform badly (e.g., “1 in x”).

This primer of risk communication for PtDA developers aims to provide a set of guiding principles based on the current evidence-base in this field. However, one of the challenges for PtDA developers is keeping up with this rapidly changing evidence base. The ongoing commitment to updates within the IPDAS Collaboration’s standards development process is one promising way of maintaining clear guidance regarding evidence-based communication approaches, but the IPDAS Collaboration faces the same sustainability challenges faced by many other international collaborative groups: it is always harder to keep a guidance document up to date than it is to create one in the first place. Nevertheless, the key approaches outlined in this paper should provide a “roadmap” for good risk communication in PtDAs and, indeed, in clinical practice more generally.

Given the fast pace of knowledge acquisition in this area, it is often difficult to identify which practices are “best.” Yet, the same knowledge base is increasingly able to identify communication practices which are known to cause problems and biases. We urge all PtDA developers and health practitioners who must communicate probabilistic risk information to patients to become aware of these adverse practices and to make sure they are not carried forward simply because of familiarity or past use.

Our work has highlighted some important gaps in the evidence base for risk communication, particularly in tailoring information, formats for conveying outcomes over time, conveying uncertainty, and the impact of interactive web-based formats. Other under-developed areas of research evidence include optimal formats for people with lower numeracy and graph literacy, and risk perceptions in different cultural contexts. Addressing these needs will require not merely targeted research efforts from selected investigators, but also a broad willingness of PtDA developers to embed experimental tests of different communication designs in their products. Only through a process of systematic, widespread investigation of risk communication methods and broad dissemination of the subsequent results can we hope to ensure that future PtDAs will present probabilistic information to patients in ways that are truly meaningful and useful to them as they make their medical decisions.

## Conclusion

Presenting numeric outcomes of decisions is an important component of good PtDA design and improves patients’ accuracy of risk perception. Good evidence exists for strategies that can improve risk communication. Our guiding principles for numeric estimates aim to assist PtDA developers to create better tools for informed decision making. Adherence to the basic principles outlined herein should be considered a quality standard for PtDAs and related risk communication materials.

## Competing interests

Lyndal Trevena, Brian Zikmund-Fisher, Mirta Galesic, Wolfgang Gassmeier, and Suzanne Linder have received in the past 5 years research funding from what is now the Informed Medical Decisions Foundation, a not-for-profit (501 (c)3) private foundation (http://www.informedmedicaldecisions.org). The Foundation develops content for patient education programs. The Foundation has an arrangement with a for-profit company, Health Dialog, to co-produce these programs. The programs are used as part of the decision support and disease management services Health Dialog provides to consumers through health care organizations and employers.

Adrian Edwards, Paul Han, John King, Margaret Lawson, Isaac Lipkus, Elissa Ozanne, Ellen Peters, Danielle Timmermans and Steve Woloshin have no conflicts to declare.

## Authors’ contributions

LT worked with BZ-F to coordinate the process and method with co-authors to develop this paper. She was co-author with MG on the section about presenting the chance of an event and was co-author with AE on the section about screening and test outcomes. She wrote the first draft of the background, methods and discussion sections of this manuscript and developed Tables 1 & 2. She coordinated comments and revisions from co-authors and finalised the referencing and other documentation for submission.

BZ-F worked with LT to identify members of the team and to coordinate the development of the paper and communications with co-authors. He was co-author with DT on the section about presenting changes in numeric outcomes and with EO on the section about interactive formats. He developed Table S1, provided initial feedback to LT in the writing of the background, methods and discussion sections, and provided comments and revisions regarding the full manuscript.

AE was co-author with LT on the section about presenting outcome estimates in screening and testing decisions, and with DT on the section about tailoring estimates. He also provided comments and revisions throughout the manuscript.

WG was co-author with IL on the section on visual formats. He also provided comments and revisions throughout the manuscript.

MG was co-author with LT on the section about presenting the chance of an event and was coauthor with IL on the section about skills for understanding numerical estimates. She also provided comments and revisions throughout the manuscript.

PH was co-author with MLL on the section about conveying uncertainty and with JK on the section about formats for understanding outcomes over time. He also provided comments and revisions regarding the full manuscript.

MLL was a member of the writing group on the section about conveying uncertainty. She contributed to editing and revisions of the full manuscript.

IL helped to review paper and produce context materials for visual communication of risk and numeracy.

SKL was co-author with JK on the section about narrative methods for conveying the chance of an event. She also provided comments and revisions regarding the full manuscript.

EP was co-author with SW on the section concerning numeric estimates in context and with evaluative labels. She also provided comments and revisions throughout the manuscript.

SW was co-author with EP on the section concerning numeric estimates in context and with evaluative labels. He also provided comments and revisions throughout the manuscript.

EO was co-author with BZ-F on the section about interactive formats. She also provided comments and revisions throughout the manuscript.

DT was co-author with BZ-F on the section about presenting changes in numeric outcomes and with AE on the section about tailoring estimates. She also provided comments and revisions throughout the manuscript.

JK was co-author with PH on the section about formats for understanding outcomes over time and with SKL on the section about narrative methods for conveying the chance of an event. He reviewed the full manuscript.

## Supplementary Material

Additional file 1 **Table S1:** Key Messages for Presenting Quantitative Information about Decision Outcomes Click here for file

## Declarations

Publication of this supplement was partially funded by an unrestricted grant from the Informed Medical Decisions Foundation. Lyndal Trevena contributed the remaining funds. Administrative and editorial support for this supplement was also provided by The University of Texas MD Anderson Cancer Center.

This article has been published as part of *BMC Medical Informatics and Decision Making* Volume 13 Supplement 2, 2013: The International Patient Decision Aid Standards (IPDAS) Collaboration’s Quality Dimensions: Theoretical Rationales, Current Evidence, and Emerging Issues. The full contents of the supplement are available online at http://www.biomedcentral.com/bmcmedinformdecismak/supplements/13/S2.
