# Supplementary material for: Presenting quantitative information about decision outcomes: a risk communication primer for patient decision aid developers
Source: BMC Med Inform Decis Mak. 2013 Nov 29;13(Suppl 2):S7. doi: 10.1186/1472-6947-13-S2-S7 (PMC4045391; doi:10.1186/1472-6947-13-S2-S7)
Supplement: Additional file 1 — Table S1: Key Messages for Presenting Quantitative Information about Decision Outcomes [file 1472-6947-13-S2-S7-S1.pdf]

**Table S1: Key Messages for Presenting Quantitative Information about Decision Outcomes**

| Communication Issues<br>In Presenting Quantitative Information | Key Messages |
|---|---|
| Presenting the Chance an Event Will Occur | • Ideal formats depend on the task that the recipient faces.<br>• Use simple frequency (e.g., x in 100) or simple percentage (e.g., x%) formats that explicitly specify the reference class over time.<br>    o Using both formats together does not appear to provide benefits.<br>• When comparing two independent events, the simple percentage format appears to be better understood than the simple frequency format, possibly because fewer numbers are simpler to process.<br>    o Specifying the reference class over time is essential.<br>    o Format biases may exist with very small numbers and for the less numerate. These may be partly corrected by use of appropriate visual display formats (see Section 6).<br>    o Use consistent denominators with simple frequency formats (i.e., no “1-in-X” formats”). |
| Presenting Changes in Numeric Outcomes | • Use absolute risk presentations (either simple frequencies or percentages) rather than relative risk presentations (e.g. “30% lower risk”), as the latter tend to magnify risk perceptions and decrease understanding.<br>    o Maintain constant denominators across statistics.<br>• Incremental risk formats (absolute risk increase or decrease) may be valuable if accompanied by visual displays. |
| Outcome Estimates for Tests and Screening Decisions | • Use of “natural frequencies” (frequency representations that use a common, fixed reference class of cases) can improve peoples’ understanding and estimates of joint occurrence risks (e.g., the probability of having breast cancer given an abnormal mammography result).<br>    o Representations of the calculated “post-test probability” may be communicated as percentages if that simplifies the user’s task. |

| | |
|---|---|
| Numerical Estimates in Context and with Evaluative Labels | • Contextual data (e.g., providing the risk of conditions other than the target condition) can help users get perspective on their risk of disease.<br>  ○ Providing such data should be considered when feasible.<br>• Directly interpreting the meaning of risk data (e.g., by providing evaluative labels such as “poor”) has a substantial impact on people’s reactions.<br>  ○ Because the appropriateness of such reactions varies, evaluative labels should be applied carefully. |
| Communicating Uncertainty | • Care should be taken to distinguish between the randomness of future events and “ambiguity” (a lack of knowledge needed to predict the likelihood of future outcomes).<br>• Many people exhibit “ambiguity aversion,” avoiding decision making and showing affective responses to situations described as having epistemic uncertainty.<br>• Little consensus exists regarding how best to communicate these concepts. |
| Visual Formats | • Visual displays such as pictographs/icon arrays and bar charts can improve understanding, especially among the less numerate<br>  ○ People vary in their graph literacy, i.e., their ability to extract data and meaning from visual displays.<br>• Visual displays convey essential or “gist” information more than precise information.<br>  ○ Bars and pictographs are perceived most accurately and easily, especially when they depict the part-whole relationship by showing the entire population. |
| Tailoring Estimates to Individual Characteristics | • Research is mixed regarding the effect of tailoring risk information. |
| Formats for Understanding Outcomes Over Time | • Efforts to estimate risk over time are often hampered by a lack of data.<br>• Multiple approaches can show risk over time, including chance of a specific outcome at a single point in the future, mortality or survival graphs, and lifetime risk estimates.<br>  ○ Research is needed to assess the relative strengths and weaknesses of different approaches |
| Narrative Methods for Conveying the Chance of | • The proportion of favorable vs. unfavorable narratives can influence perceptions of |

| | |
|---|---|
| an Event | risk and treatment choices.<br>◦ When used to present risk or benefit information, they should be accompanied by a visual display such as pictographs.<br>• Narratives should be used with caution until research better clarifies their effects (both positive and negative). |
| Important Skills for Understanding Numerical Estimates | • Higher numeracy facilitates computations, interpretations of numbers, information seeking, depth of processing and, trust in numerical formats.<br>◦ Lower numeracy is associated with overestimation of risk probabilities, higher susceptibility to other factors such as format, and denominator effect.<br>• Both objective and subjective measures of numeracy are now available. |
| Interactive, Web-based Formats | • While interactive, web-based formats can use motion cues or game-like interfaces to potentially reinforce risk messages, they may degrade knowledge unless these elements reinforce the most critical gist message. |
